# GTPase Rac Regulates Conidiation, AFB1 Production and Stress Response in Pathogenic Fungus *Aspergillus flavus*

**DOI:** 10.3390/toxins14090581

**Published:** 2022-08-24

**Authors:** Ling Qin, Lan Yang, Jiaru Zhao, Wanlin Zeng, Minxuan Su, Shihua Wang, Jun Yuan

**Affiliations:** Key Laboratory of Pathogenic Fungi and Mycotoxins of Fujian Province, Key Laboratory of Biopesticide and Chemical Biology of Education Ministry, School of Life Sciences, Fujian Agriculture and Forestry University, Fuzhou 350002, China

**Keywords:** *A. flavus*, Rac, conidiation, aflatoxins, stress response

## Abstract

As a member of the Rho family, Rac plays important roles in many species, including proliferation, differentiation, apoptosis, DNA damage responses, metabolism, angiogenesis, and immunosuppression. In this study, by constructing *Rac*-deleted mutants in *Aspergillus flavus*, it was found that the deletion of *Rac* gene led to the decline of growth and development, conidia production, AFB1 toxin synthesis, and seed infection ability of *A. flavus*. The deletion of *Rac* gene also caused the disappearance of *A. flavus* sclerotium, indicating that *Rac* is required for sclerotium formation in *A. flavus*. The sensitivity of *Rac*-deficient strains responding to cell wall stress and osmotic pressure stress increased when compared to *A.* *flavus* WT. The Western blot result showed that mitogen-activated serine/threonine-protein kinase Slt2 and mitogen-activated protein kinase Hog1 proteins were no longer phosphorylated in *Rac*-deficient strains of *A. flavus*, showing that Rac may be used as a molecular switch to control the Slt2-MAPK cascade pathway and regulate the osmotic Hog-MAPK cascade pathway in *A. flavus* in response to external stress. Altogether, these results indicated that Rac was involved in regulating the growth and development, conidia formation and AFB1 synthesis, and response to cell wall stress and osmotic pressure stress in *A. flavus*.

## 1. Introduction

*Aspergillus flavus*, as a common saprophytic filamentous fungus, is widely distributed all over the world. It is not only a plant pathogenic fungus, but also a conditional human and animal pathogenic fungus. Many secondary metabolites are produced during the growth and development of *A. flavus*, among which aflatoxins are produced from polyketones [1]. *A. flavus* produces aflatoxins by infecting cereal crops, including corn, peanut, and wheat [2], which causes a variety of harm to humans and animals, such as hepatotoxicity [3], carcinogenicity [4] and immunotoxicity [5]. Aflatoxin-contaminated cereal crops pose serious health risks in developing countries [6], as well as significant economic losses to the United States and other developed countries [7]. Therefore, the study of *A. flavus* is of more practical significance under the current situation of food security and shortage in many countries.

Rho family proteins belong to one of the important members of the Ras superfamily, including the Rho, Rac, and Cdc subfamilies. Members of this family have the inherent activity of hydrolyzing GTPases, so they are accustomed to being called Rho GTPases. As a molecular switch, Rho GTPases almost participate in the basic cellular process. The GTP binding state is activated, and the GDP binding state is inactivated [8]. Rho GTPase can regulate actin cytoskeleton [9], cell cycle progression [10], and signal network of gene expression, thus regulating normal cell proliferation, differentiation and apoptosis, and is closely related to tumor occurrence and metastasis.

As one of the most studied proteins in the Rho family, Rac plays an extremely important role in many species. As early as 1955, Rac was proved to regulate the production of reactive oxygen in macrophages in response to the attack of pathogens [11]. In *Drosophila melanogaster*, Rac not only plays an important role in neuron development [12] but also participates in axon growth and myoblast fusion [13]. Rac seems to activate LIM kinase to inhibit axon growth via the effector kinase PAK, but may also activate a PAK-independent pathway that promotes axon growth [12,14]. In fungi, such as *Aspergillus nidulans*, *Rac*-deficient mutants showed slow growth and development, but there is no obvious difference in mycelium morphogenesis. However, the conidia had various defects, such as the appearance of vesicles without conidia, shortening of stalk and vesicle branches, irregular shape of vesicles, and reduction in the number of conidia and vesicles [15]. The *RacA* gene was found to play an important role in the growth and pathogenesis of *Aspergillus* *fumigatus*. The absence of *RacA* in *A.* *fumigatus* reduced the production of reactive oxygen but had little effect on virulence [16]. In dimorphic plant pathogenic fungus *Ustilago maydis*, it was found that *Rac1* deletion resulted in the loss of virulence, affecting cell morphology and interfering with mycelium growth [17]. Unlike the other Rho GTPases, Rac orthologs were not found in unicellular yeasts such as *Saccharomyces cerevisiae* and *Schizosaccharomyces pombe*, suggesting that the *Rac* gene might have evolved with the increase in cell complexity [18]. However, the function of Rac in *A. flavus* has not been explored until now. Therefore, this paper focused on the Rac biofunctions in *A. flavus*, and the main results were shown as follows.

## 2. Result

### 2.1. Biological Analysis of Rac

**Figure 1:**
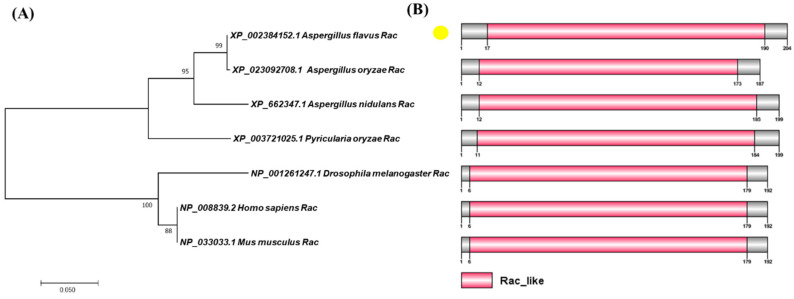
Bioinformatics analysis of Rac protein. (**A**) The phylogenetic relationship of Rac in selected eukaryotes was analyzed and visualized by MEGA7.0 software (Mega Limited, Auckland, New Zealand). (**B**) The domain of Rac protein was identified and visualized by SMART/(http://smart.embl-heidelberg.de) and DOG2.0 software (Jian Ren, Hefei, China).

Blast comparison was carried out with *A. nidulans* Rac protein (XP_662347.1) in NCBI to identify homologous protein AflRac (XP_002384152.1) in *A. flavus*. Then, AflRac was compared with Rac proteins of other four fungi to study whether Rac proteins from fungi are conserved. Since Rac protein also plays an important role in the development of animal nerves, we compared Rac homologous proteins in two vertebrates. We used MEGA7 to map the phylogenetic tree based on the protein sequences blasted in NCBI. The result showed that Rac protein sequences are highly homologous in fungi and vertebrates (Figure 1A), indicating that the Rac protein is highly conserved in the process of evolution. We used DOG2.0 software (Jian Ren, Hefei, China) to draw the domain diagram of the proteins mentioned above and found that all these proteins have a Rac-like domain (Figure 1B), indicating that the Rac protein domain is also highly conserved in these species.

### 2.2. Rac from A. flavus Is a GTPase

**Figure 2:**
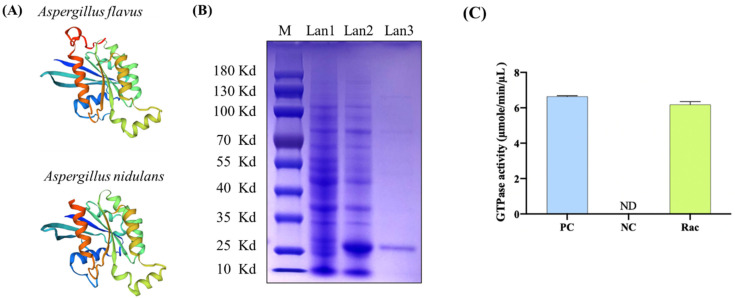
Expression and GTPase activity assay. (**A**) *A. flavus* Rac (XP_002384152.1) sequence is similar to *A. nidulans* Rac (XP_662347.1; 90.69% identify), and they had highly conserved three-dimensional protein structure. (**B**) SDS-PAGE assay of the expressed recombinant protein and purification result. Line 1: the expressed recombinant plasmid pET28a-*Rac* without IPTG, line 2: the expressed recombinant plasmid pET28a-*Rac* with IPTG induction, line 3: the purified recombinant protein. (**C**) GTPase activities of Rac protein. PC = positive control, NC = negative control.

After systematic prediction, sequence alignment showed that *A. flavus* Rac (XP_002384152.1) had high homology with *A. nidulans* Rac (XP_662347.1, 90.69% identification) and they two showed highly conserved three-dimensional protein structures (Figure 2A). In order to confirm whether AflRac has GTPase activity, we cloned the coding sequence of AflRac using the cDNA of *A. flavus* wild type (WT) as template and ligated it into the pET28a vector. After sequencing and twice transformation, the Rac protein expression strain was successfully constructed. After induction by IPTG, it was purified by a Ni^2+^-NTA column (Figure 2B). GTP enzyme activity was detected *in vitro*, and it was found that the AflRac protein had GTP enzyme activity (Figure 2C). The above results proved that AflRac has GTPase activity in *A. flavus*. Thus, in this paper, we directly define the protein XP_002384152.1 in *A. flavus* as Rac.

### 2.3. Rac Is Involved in Vegetative Growth and Conidiation

**Figure 3:**
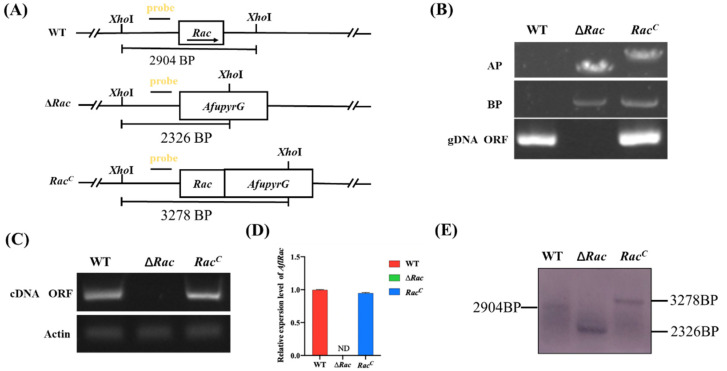
Generation of deletion and complementation strains. (**A**) The deletion mutant was constructed by homologous recombination. (**B**) The construction was verified by routine PCR with gDNA as template. (**C**) RT-PCR was used to verify the construction with cDNA as template. (**D**) The expression level of *Rac* gene was verified by qPCR. (**E**) Southern blot proved that ∆*Rac* strain and strain *Rac^C^* were successfully constructed. The length of the WT fragment cut out by *Xho*I was 2904 bp, ∆*Ra*c strain was 2326 bp, and *Rac^C^* was 3278 bp.

**Figure 4:**
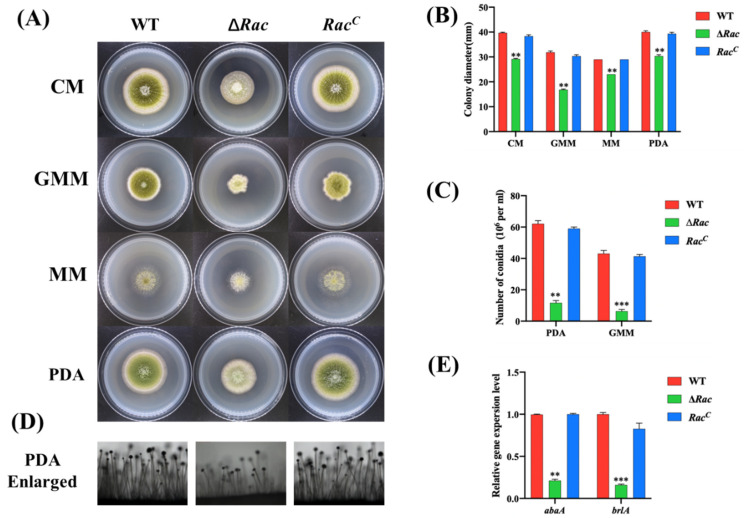
*Rac* was involved in vegetative growth and conidiation in *A. flavus.* (**A**) WT, ∆*Ra*c and *Rac*^C^ were cultured in two kinds of rich media (CM, PDA) and two kinds of basic media (GMM, MM) at 37 °C for 3 days. (**B**) GraphPad Prism8 was used to analyze the growth diameter of colonies. (**C**) Spores number of these strains on the rich medium (PDA) and basic medium (GMM). (**D**) Amplification of conidiophore cultured on PDA medium. (**E**) qPCR verified the expression level of genes *abaA* and *brlA* in these strains. ** mean *p* < 0.05, *** means *p* < 0.0001.

To study the function of Rac in *A. flavus*, we constructed *Rac-*deleted mutant strains. Using the principle of gene recombination, we replaced *Rac* in *A. flavus* wild type with *AfupyrG* to construct *Rac* deletion mutants (∆*Rac*) (Figure 3A). At the same time, we constructed *Rac* complementary strains (*Rac^C^*). Then, PCR (Figure 3B), RT-PCR (Figure 3C), qPCR (Figure 3D), and Southern blot (Figure 3E) results verified that ∆*Rac* and *Rac^C^* were successfully constructed. The above-constructed strains were diluted to the same multiple and inoculated in two kinds of rich media (CM and PDA) and two kinds of basic media (GMM and MM) for culture (Figure 4A). By observing the colony growth, it was found that the diameter of ∆*Rac* in all media was significantly smaller than that of WT and *Rac^C^* (Figure 4B), which indicates that Rac participates in the vegetative growth process of *A. flavus*. During the observation, we also found that the color of ∆*Rac* was obviously white, as the green color on the strains was conidia of *A. flavus*. Therefore, a rich medium (PDA) and a basic medium (GMM) were selected to culture all the above strains, and the conidia were eluted to count the number with a blood cell counter under an optical microscope. The results showed that the conidia production of ∆*Rac* was significantly lower than that of WT and *Rac^C^* (Figure 4C). The decrease in conidia made us wonder whether it was related to the decrease in conidiophore. Therefore, we observed the conidiophore and found that the number of conidiophore of ∆*Rac* was significantly decreased than that of WT and *Rac^C^* (Figure 4D), indicating that Rac was very important for the formation of conidiophores of *A. flavus*. We also verified the key genes related to conidia formation in *A. flavus* by qPCR and found that the expression levels of genes *abaA* and *brlA* were significantly decreased in ∆*Rac* (Figure 4E), which further indicated that Rac regulates the expression of genes related to conidiation, thus affecting the conidia formation of *A. flavus*.

### 2.4. Rac Is Required for Sclerotial Formation in A. flavus

**Figure 5:**
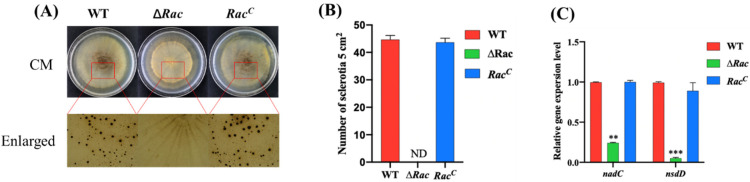
*Rac* is required for sclerotial formation in *A*. *flavus.* (**A**) The strain was cultured in CM medium at 37 °C for 7 days and then observed. (**B**) Number of sclerotia of these strains. (**C**) qPCR verified the expression level of genes *nsdC* and *nsdD* in these strains. ** means *p* < 0.05, *** means *p* < 0.0001.

Sclerotium is another form of *A. flavus* reproduction, so the effect of Rac on the sclerotium formation of *A. flavus* deserves our study. We cultured the strains in CM medium for 7 days to observe the sclerotium formation, and the results showed that the ∆*Rac* did not produce sclerotium at all, but WT and *Rac^C^* of *A. flavus* produced normal sclerotium (Figure 5A,B). qPCR result showed that the expression of *nadC* and *nsdD*, the key genes of sclerotium formation, was decreased markedly in ∆*Rac* when compared to WT and *Rac^C^* (Figure 5C), which indicated that *Rac* was involved in sclerotium formation in *A. flavus*. This implies that Rac may act as a molecular switch to regulate the expression of genes related to the sclerotium formation pathway. When *Rac* was deleted, the sclerotium formation process was blocked and sclerotium disappeared due to the lack of control switch in *A. flavus*. All the above results showed that Rac is involved in the development and reproduction of *A. flavus*.

### 2.5. Effect of Rac on Aflatoxin Biosynthesis in A. flavus

**Figure 6:**
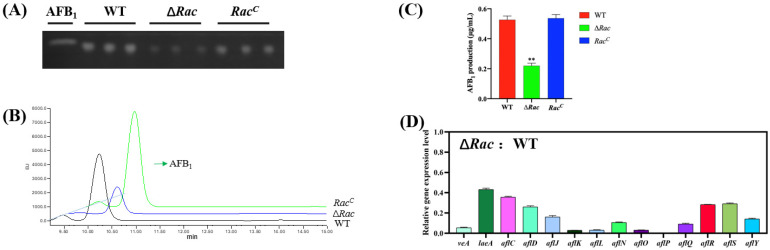
Effect of *Rac* on aflatoxin biosynthesis in *A. flavus.* (**A**) TLC was used to observe AFB1 production of the strains. (**B**) Determination of AFB1 production by HPLC. (**C**) According to the HPLC data, the amount of AFB1 produced in the strains was calculated, and the histogram was drawn and analyzed by GraphPad Prism8. ** means *p* < 0.05. (**D**)The expression level of toxin synthesis-related and regulatory genes in ∆*Ra*c was calculated, and the histogram was drawn and analyzed by GraphPad Prism8.

AFB1 is the most harmful secondary metabolite produced by *A. flavus*. To study the role of Rac in AFB1 production, all strains were cultured in liquid medium, and TLC and HPLC were used to detect the aflatoxin production. The result showed that the ability of *Rac* mutant to produce toxin AFB1 was impaired significantly compared to WT and *Rac^C^* (Figure 6A–C). To explore the reason for the significant decrease of AFB1 in ∆*Rac*, we amplified secondary metabolism-related genes and regulatory genes in the aflatoxin formation gene cluster by qPCR. It was found that the expression of secondary metabolism regulatory genes *veA* and *laeA* was decreased, and the expression of genes in the toxin formation gene cluster (*aflR*, *aflC*, *aflD*, *aflJ*, *aflK*, *aflL*, *aflN*, *aflO*, *aflP*, *aflQ*, *aflR*, *aflS*, *aflY*) was also downregulated (Figure 6D). It could be speculated that Rac might regulate the expression of genes related to toxin synthesis, thereby affecting the toxin synthesis ability in *A. flavus*.

### 2.6. Role of Rac in Seed Infection

**Figure 7:**
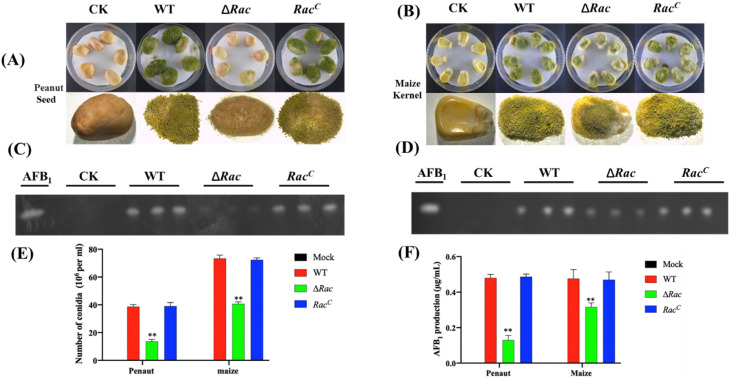
Role of Rac in seed infection. (**A**) Peanut was infected with uniformly diluted 1 × 10^6^ conidia solution and cultured at 29 °C for 7 days. (**B**) The corn was infected with the uniformly diluted 1 × 10^6^ conidia solution and cultured at 29 °C for 7 days. (**C**) Detection of aflatoxin production by infected peanut by TLC. (**D**) Detection of aflatoxin production by infected corn by TLC. (**E**) Spore number from infected peanut and corn. (**F**) Amount of toxin produced by infected peanut and corn. ** means *p* < 0.05.

In order to explore the ability of ∆*Rac* to infect seeds and produce aflatoxin in the process of infecting the host, we conducted an in vitro infection experiment. The same amount of conidia was inoculated to corn and peanut seeds and cultured for 7 days, and it was found that the conidia produced by ∆*Rac* in the process of infecting seeds decreased significantly when compared to WT and *Rac^C^* (Figure 7A,B,E), and the toxins produced by ∆*Rac* in the infecting process were significantly reduced when compared to WT and *Rac^C^* (Figure 7C,D,F). All the above results showed that *Rac* also played an important role in the process of *A. flavus* infecting seeds.

### 2.7. Rac Contributes to Cell Wall and Osmotic Stress Response

**Figure 8:**
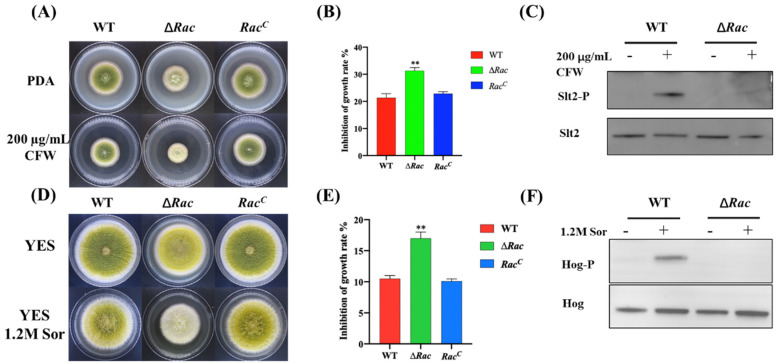
*Rac* contributes to cell wall stress and osmotic stress. (**A**) CFW was added to PDA culture medium, and strains with the same dilution ratio were cultured at 37 °C for 3 days. (**B**) The inhibition rate was calculated by the ratio of the absolute value of the growth diameter of the stressed strain to that of the unstressed strain. (**C**) Western blot assay of Slt2 and Slt2-P proteins in WT and ∆*Ra*c were studied by Slt2- and Slt2-P-specific antibodies. (**D**) Sorbitol was added into PDA culture medium and cultured at 37 °C for 3 days. (**E**) The inhibition rate was calculated in these strains. (**F**) Western blot of Hog and Hog-P proteins in WT and ∆*Ra*c by using Hog and Hog-P-specific antibodies. ** means *p* < 0.05.

It was found that when 1.2 M calcofluor white (CFW) was added to the culture medium (Figure 8A), the inhibition rate of cell wall stress to ∆*Rac* was higher than that to WT and *Rac^C^* (Figure 8B), indicating that the sensitivity of ∆*Rac* to respond to cell wall stress increased. Western blot results indicated that Slt2 could not be phosphorylated after *Rac* deletion (Figure 8C), showing that Rac may be used as the switch for the Slt2-MAPK cascade in *A. flavus* to control the pathway. When 1.2 M sorbitol was added into the culture medium (Figure 8D), the results showed that the inhibition rate of ∆*Rac* was also increased when compared to WT and *Rac^C^* (Figure 8E), indicating the sensitivity of ∆*Rac* responding to osmotic stress was increased. Western blot results found that Hog could not be phosphorylated after *Rac* was deleted (Figure 8F), which indicated that Rac could be used as the switch of the osmotic glycerol pathway in *A. flavus* to regulate the Hog-MAPK cascade to cope with osmotic stress.

## 3. Discussion

In plants, Rac orthologs are thought to be involved in the regulation of pollen tube growth, which shares several features with filamentous growth [19]. As the first Rac homologous gene studied in fungi, the deletion of *YlRAC1* resulted in the morphological changes of dimorphic yeast *Yarrowia lipolytica* cells, suggesting that the function of YlRAC1 may be related to some aspects of cell growth polarization [20]. In filamentous fungi, it was reported that the absence of *RacA* in *Aspergillus niger* leads to hyper-branched reproductive tubes and hyphae, which are short in length but wide in hyphal diameter. The frequent branching leads to tighter colonies on solid media, and the diameter of colonies becomes smaller due to the slow elongation at the tip [21]. In this study, we found that in *A. flavus*, when *Rac* was absent, the elongation rate of the apical hypha was low, the colony diameter was obviously reduced, and the strain morphology and development defects were serious, which was very similar to the phenotype of *A. niger*. Therefore, Rac in *A. flavus* may affect the hypha extension by acting on cell polarization and finally, affect the colony diameter.

Both *A.* *nidulans* and *A.* *fumigatus*, which are currently known to be sequenced, have been found to contain a plethora of clustered genes specifically for the production of secondary metabolites [22]. In *A. nidulans*, *aflR* and *stcU* were not expressed when *laeA* was deleted. However, when *aflR* is deleted, it does not affect the transcription level of *laeA*. When *aflR* is overexpressed, the expression of *laeA* decreases. This indicates that *laeA* can regulate the key gene *aflR* in the production of aflatoxins [23]. In *A. parasiticus* and *A. flavus*, *veA* was necessary for the transcription of the key genes of toxin production *aflR* and *aflJ* [24,25]. In this study, we found that the expression level of *veA* and *laeA* in ∆*Rac* was decreased compared with WT. The expression of the AFB1 synthesis gene cluster was decreased, and the AFB1 production ability of ∆*Rac* also declined. Therefore, we suspect that Rac may regulate the key genes *aflR* and *aflS* to control the other toxin production gene cluster to participate in toxin synthesis. It can also regulate the expression level of *laeA* and *veA*, which are involved in the switch toward secondary metabolism, thus regulating aflatoxin synthesis.

It is known in previous reports that not only aflatoxin production is regulated by veA, but also *veA* is a necessary gene for sclerotium formation in *A.* *flavus* [26]. In this study, it was found that ∆*Rac* did not produce sclerotium. The qPCR results also showed that compared with WT, the expression of *veA* of ∆*Rac* was extremely low, which was consistent with the previously reported results. It is not difficult to speculate that Rac may control the expression of *veA* in *A. flavus*, thus affecting sclerotium formation.

Compared with *A. fumigatus*, the virulence of RacA-deleted mutant is unchanged [16], but in *U.maydis*, the deletion of Rac1 leads to the loss of virulence [17]. In *Claviceps purpurea*, it was found that after *Rac* was deleted, its pathogenicity was completely lost [27]. The *Rac*-deleted mutant cannot penetrate the plant surface, which due to the serious cytoskeleton defects of the mutant, thus it cannot produce the mechanical force required for penetration. Unlike the absence of *RacA* in *A. fumigatus* [16], we found that the infectivity of *A. flavus Rac*-deleted mutant was greatly reduced compared with that of WT. Therefore, we suggested that when Rac is absent, the mutant strains have serious cytoskeleton defects, and the mechanical force required for penetration in *A. flavus* is low, which leads to the decline of infection ability.

It has been reported that under high osmotic pressure, Rac-induced activation of the PAK2 subtype leads to its phosphorylation and translocation to the intercellular junction, where it locally promotes the phosphorylation of MLC [28], which is a cascade reaction of cells to external osmotic pressure. According to our research results, the absence of Rac made Slt2 and Hog no longer phosphorylated, and the sensitivity of the strain to cope with cell wall stress and osmotic stress increased. It could be concluded that Rac is indeed involved in the pathway of *A. flavus* responding to external cell wall stress and osmotic stress. Combining with the reports [29], we speculated that Rac may play its molecular switch function as a member of the Rho family, and control the phosphorylation process of Slt2 and Hog, thus opening the communication path of the strain against environmental stress.

## 4. Materials and Methods

### 4.1. Strains and Media

All strains used in the experiment are listed in Table 1. *A. flavus* CA14 PTS (∆*ku70*∆*pyrG*, uracil auxotrophic) was used as the parent strain for transformation. The strains in this study were cultured on yeast extract sucrose (YES), minimal medium (MM), potato dextrose agar (PDA), glucose minimal medium (GMM), and complete medium (CM) for conidia culture and mycelial growth [30]. The sclerotia-inducing Wickerham (WKM) medium was used for sclerotia formation. YES liquid medium was used for AFB1 production. Each experiment was repeated at least 3 times.

### 4.2. Domain and Phylogenetic Tree Analysis

The sequence of *A. flavus* Rac (XP_002384152.1) was downloaded from NCBI and compared to the gene sequences of human, mouse, and other fungi. The phylogenetic tree was established with MEGA 7.0 software [31]. The protein domain was predicted by SMART [32] (http://smart.embl-heidelberg.de/) software and plotted by DOG2.0 software [33].

### 4.3. Expression of Recombinant Protein and Detection of GTPase Activity

The Rho GTPase *Rac* gene was cloned by PCR using special primers which include restriction sites of *EcoR* I and *Hind* III. The pET28a vector and *Rac* gene fragment were digested by *EcoR* I and *Hind* III, and then ligated by T4 ligase. Positive plasmid pET28a-*Rac* was extracted and transformed into an expression strain of *E. coli* Rosetta, grown in liquid LB medium, and further incubated at 37 °C for 4–5 h until the OD value reached 0.5. Isopropyl-β-D-thiogalactopyranoside (IPTG, 0.4 mM) was added into the medium for inducing target protein expression. After 4 h induction, cells were collected and then sonicated on ice. Cell lysates were centrifuged at 13,000 r/min until no significant precipitation. The supernatant was loaded into a Ni^2+^-NTA column for purification [34]. GTPase activities were measured using the ATPase/GTPase Activity Assay Kit (MAK-113, Sigma-Aldrich, St. Louis, MO, USA) according to the manufacturer’s instructions.

### 4.4. Construction of Mutant Strains

The *Rac* gene deletion mutants (∆*Rac*) were constructed by homologous recombination [35]. The primers used in this study were listed in Table 2. PCR amplification was used to generate a gene deletion cassette, and the PCR product was transformed into *A. flavus* CA14 protoplast. Positive transformants were screened and verified by diagnostic PCR [36] and Southern blot. Then, the two-step method was used to construct the complementary strains (*Rac^C^*) [37], and positive transformants were verified by PCR verification.

### 4.5. Mycelial Growth, Conidiation and Sclerotia Analysis

The phenotypes of WT, ∆*Rac*, and *Rac^C^* strains were observed and analyzed in different media. The mycelium growth and development, conidia formation, and sclerotium formation were analyzed according to the methods described previously [38].

### 4.6. Aflatoxins Analysis

In order to produce aflatoxin, 10 μL conidia suspensions of WT, ∆*Rac*, and *Rac^C^* strains (1 × 10^7^ conidia/mL) were inoculated into 7.5 mL YES liquid medium, respectively, and cultured at 29 °C in the dark for 7 days. Thin-layer chromatography (TLC) was used to detect aflatoxin products [39]. All the culture toxin medium was transferred to a 50 mL centrifuge tube, and an equal volume of chloroform was added and mixed well. After shaking and extraction for 30 min, the lower layer of chloroform was taken and volatilized. The mycelium was filtered and dried to obtain the dry weight of mycelium. Chloroform lytic toxin was added according to the proportion of dry weight of mycelium. TLC developing agent (volume ratio, chloroform: acetone = 9:1) was poured into the chromatography cylinder, and then 10 µL of each sample was added to the TLC plate. After completion, the samples were air-dried and placed under the UV gel imaging system for toxin detection. In order to quantitatively analyze the yield of aflatoxin, the aflatoxin extract was further analyzed by high-performance liquid chromatography (HPLC). Aflatoxin was detected by fluorescence detector (Water, Milford, MA, USA) with excitation wavelength and emission wavelength of 365 nm and 455 nm, respectively. Each experiment was repeated 3 times [29].

### 4.7. Seeds Infections

The ability of WT, ∆*Rac*, and *Rac^C^* strains to infect plant seeds was tested by previous methods [40]. Seeds were inoculated with conidia suspension and cultured at 29 °C; then, 700 μL of sterile water was added to keep the culture filter paper in a moist state. After 6 days of culture, the infected seeds were collected and placed into a 50 mL centrifuge tube, and then 15 mL sterile water and 7.5 mL dichloromethane were added. In order to make the conidia fully suspend in the liquid, the tube was shaken violently for 5 min. The number of conidia was counted, and aflatoxin was extracted according to the previous method [41]. Each experiment was repeated at least 3 times.

### 4.8. Cell Wall Stress and Osmotic Pressure Stress

Two hundred µg/mL CFW was added to PDA medium to construct cell wall stress medium, and 1.2 mol/mL sorbitol was added to YES medium to construct osmotic stress medium. The strains with the same dilution ratio were cultured at 37 °C for 3 days [42].

### 4.9. Western Blot Analysis

The conidia (6 × 10^5^) of WT, ∆*Rac* and *Rac^C^* strains were inoculated into YES liquid medium, respectively, and cultured for 48 h. Whole-cell extraction and Western blot were carried out according to our previous publication [43]. Anti-Hog1 (Santa Cruz Biotechnology Company, Dallas, TX, USA) and anti-AflSlt2 antibodies (prepared by our laboratory) were used. Enhanced chemiluminescence (ECL) substrate was used for Western blot analysis, and chemiluminescence was determined by G-box Chemi XT4 (Syngene, Hong Kong, China).

### 4.10. Quantitative RT-PCR Analysis

The mycelia of WT, ∆*Rac*, and *Rac^C^* strains were harvested on YES medium at 37 °C. Total RNA of mycelium was extracted by an RNA extraction kit (Tianmo Bio, Beijing, China), and reverse transcription cDNA was obtained by gene synthesis SuperMix (Transgen Biotech, Beijing, China). On the ThermoFisher Scientific real-time PCR system, SYBR Green qPCR Mix (Guangzhou Dongsheng Biotechnology, Guangzhou, China) was used for qRT-PCR. The *actin* gene of *A. flavus* was used as the reference gene, and the relative expression of the target gene was calculated by the 2^-^^∆∆Ct^ method [44]. The qRT-PCR primers were listed in Table 3. All qRT-PCR tests were performed in triplicate for each sample, and each experiment was repeated at least 3 times.

### 4.11. Statistical Analysis

GraphPad Prism8 [45] (https://www.graphpad.com) was used for statistical analysis. T-test was used for comparison between the two groups, and ANOVA was used for comparison among multiple groups.

## Figures and Tables

**Table 1:** Strains used in this study.

Strains	Genotype Descripion	Source
*A. flavus* CA14	Δ*ku70*, Δ*pyrG*	Purchased from FGSC (Manhattan, NY, USA)
Wild-type (WT)	Δ*ku70*, Δ*pyrG*::*AfpyrG*	This study
Δ*AflRac*	Δ*ku70*, Δ*pyrG*::*AfpyrG*, Δ*AflRac*	This study
Δ*AflRacC*	Δ*ku70*, Δ*Aflmsb2*:: *Aflmsb2*, *pyrG*	This study
*E. coil* DH5*α*		Takara
*E. coil* Rosetta		Takara

**Table 2:** Primer*s* used in this study.

Primer	Sequence (5′-3′)	Characteristics
*Rac*-p1	GGTTTCCTCAACGGTGTT	For amplifying
*Rac*-p3	GGGTGAAGAGCATTGTTTGAGGC TCTTTCAGAATCTGCGATAT	5′UTR of Δ*Rac*
*Rac*-p6	GCATCAGTGCCTCCTCTCAGA CAATTTTCTCCCGACTATAA	For amplifying 3′ UTR of Δ*Rac*
*Rac*-p8	CATCATTCCTAATGTGCTT	
*Rac*-p2	GTTGGGAAAGAGGTGTCG	For fusion PCR
*Rac*-p7	GTCTCAGTGCGTGTTGCT	of Δ*Rac*
*pyrG*-F	GCCTCAAACAATGCTCTTCACCC	For amplifying
*pyrG*-R	GTCTGAGAGGAGGCACTGATGC	*A. fumigatus* pyrG
*Rac*-p9	CGGCTAATAGACGACCAAT	For validating ORF
*Rac*-p10	AGACGCTCTTCAGATTACG	
*Rac*-C-p1	GATTGTTCCCTTATCATTG	For amplifying
*Rac*-C-p2	CGAACAAGGTGTATAGTCT	ORF of *RacC*
*Rac*-C-p3	GATTGTTCCCTTATCATTG	
*Rac*-C-p4	GGGTGAAGAGCATTGTTTGAG GCCTACAGAATCAGACATTTGCTCTTC	For amplifying 5′UTR of *Rac^C^*
*Rac*-C-p5	GCATCAGTGCCTCCTCTCAGA CATCGATTCTTATAATTTTCTCCCGA	For amplifying 3′UTR of *Rac^C^*
*Rac*-C-p6	CGAACAAGGTGTATAGTCT	
*Rac*-C-O1	CCTGCCTTGTGGTATTTC	For fusion PCR
*Rac*-C-O2	ATGCTTTGCTGACGCTAT	of *Rac^C^*
*Rac*-S-O1	ACCAGCCATTCAGTGTTC	For Southern blot
*Rac*-S-O2	AATTGCAGTGACAAGAGATG	
*pyrG*-907-F	ATGACGGCGATGTAGGGA	For screening Δ*Rac* mutant
*pyrG*-919-R	CGACATCCTCACCGATTTCA	
*Rac*-G-O1	AATGGGTCGCGGATCCCTGGAAGTTC TGTTCCAGGGGCCCATGGCGACCGGT	For amplifying
*Rac*-G-O2	GGTGGTGGTGGTGGTGCTCGAGCTACAGA ATCAGACATTTGCTC	complete *Rac* gene

**Table 3:** qPCR primer*s* used in this study.

Primer	Sequence (5′-3′)	Characteristics
*abaA*-qRT-F	TCTTCGGTTGATGGATGATTTC	For amplifying *abaA*
*abaA*-qRT-R	CCGTTGGGAGGCTGGGT	
*brlA*-qRT-F	GCCTCCAGCGTCAACCTTC	For amplifying *brlA*
*brlA*-qRT-R	TCTCTTCAAATGCTCTTGCCTC	
*nsdC*-qRT-F	GCCAGACTTGCCAATCAC	For amplifying *nsdC*
*nsdC*-qRT-R	CATCCACCTTGCCCTTTA	
*nsdD*-qRT-F	GGACTTGCGGGTCGTGCTA	For amplifying *nsdD*
*nsdD* -qRT-R	AGAACGCTGGGTCTGGTGC	
*veA -qRT-F*	TATCATTCCGTGGCTCAAT	For amplifying *veA*
*veA -qRT-R*	GAGAGGTACTGCTGGATG	
*laeA -qRT-F*	TTGTTGGGGTTGACCTTGCT	For amplifying *laeA*
*laeA -qRT-R*	GCCATCCCATCACACTTCCA	
*aflC-*qRT-F	TTACGCTGCGATCAGTTCCTC	For amplifying *aflC*
*aflC-*qRT-R	CGACTCGCATTACAGCATCTAAC	
*aflD-*qRT-F	GTGGTGGTTGCCAATGCG	For amplifying *aflD*
*aflD-*qRT-R	CTGAAACAGTAGGACGGGAGC	
*aflJ-*qRT-F	CGGCGTATGAGGAGAATG	For amplifying *aflJ*
*aflJ-*qRT-R	CTTCATCAACCTGGCATCA	
*aflK-*qRT-F	GAGCGACAGGAGTAACCGTAAG	For amplifying *aflK*
*aflK-*qRT-R	CCGATTCCAGACACCATTAGCA	
*aflL-*qRT-F	GGCTGCGGAACTGTATTG	For amplifying *aflL*
*aflL-*qRT-R	TGTGGAGTGCTGGAAGAG	
*aflN-*qRT-F	TTCATTCCTGAGCGATGG	For amplifying *aflN*
*aflN-*qRT-R	CGTATGCTGGCGTAATATC	
*aflO-*qRT-F	GATTGGGATGTGGTCATGCGATT	For amplifying *aflO*
*aflO-*qRT-R	GCCTGGGTCCGAAGAATGC	
*aflP-*qRT-F	ACGAAGCCACTGGTAGAGGAGATG	For amplifying *aflP*
*aflP-*qRT-R	GTGAATGACGGCAGGCAGGT	
*aflQ-*qRT-F	GTCGCATATGCCCCGGTCGG	For amplifying *aflQ*
*aflQ-*qRT-R	GGCAACCAGTCGGGTTCCGG	
*aflR-*qRT-F	AAAGCACCCTGTCTTCCCTAAC	For amplifying *aflR*
*aflR-*qRT-R	GAAGAGGTGGGTCAGTGTTTGTAG	
*aflS-*qRT-F	AAGCTAAGGCCGAGTCTGG	For amplifying *aflS*
*aflS-*qRT-R	CAGGTTGTGTTGCTGTTGATAG	
*aflY-*qRT-F	AGGCAGACTTTCTAACACT	For amplifying *aflY*
*aflY-*qRT-R	CCTTCAGTTCCACACCAA	
*Rac* -qRT-p9	GGTGACGGTGCTGTTGGA	
*Rac* -qRT-p10	CGGGTCGTGGATTGAGAA	
*actin*-qRT-F	ACGGTGTCGTCACAAACTGG	For amplifying *actin*
*actin* -qRT-R	CGGTTGGACTTAGGGTTGATAG	

## Data Availability

Not applicable.
